# Adjunctive Greater Occipital Nerve Block for Pain Control in Medically Refractory Acute Primary Angle Closure: An Observational Study

**DOI:** 10.3390/jcm15072754

**Published:** 2026-04-05

**Authors:** Sang Yoong Park, Eun Seong Kim, Sang Wook Jin

**Affiliations:** 1 Department of Anesthesiology and Pain Medicine, Dong-A University College of Medicine, Busan 49201, Republic of Korea; 2 Department of Ophthalmology, Dong-A University College of Medicine, Busan 49201, Republic of Korea

**Keywords:** greater occipital nerve block, acute primary angle closure, ocular pain, headache, regional anesthesia, analgesia

## Abstract

**Background**: Acute primary angle closure (APAC) is an ophthalmic emergency characterized by abrupt elevation of intraocular pressure (IOP) and severe ocular pain and headache. While acute management prioritizes IOP reduction, supportive analgesic strategies during the preoperative waiting period in medically refractory cases remain insufficiently studied. We evaluated short-term changes in the pain intensity and safety of adjunctive greater occipital nerve block (GONB) in medically refractory APAC. **Methods**: This retrospective observational study included 34 patients with medically refractory APAC who received GONB during the preoperative waiting period. Headache intensity was measured using an 11-point Numeric Rating Scale (NRS) at baseline, 30 min, and 60 min. Longitudinal changes were analyzed using a linear mixed-effects model. Responder analyses were reported with 95% confidence intervals (Wilson method). No multivariable modeling or NNT estimation was performed in the revised analysis. **Results**: Baseline NRS was 7.8 ± 1.1, decreasing to 4.1 ± 1.5 at 30 min and 3.6 ± 1.3 at 60 min (both *p* < 0.001). The mean baseline-to-60 min change was −4.21 (95% CI, −4.88 to −3.54). Clinically meaningful pain relief (≥3-point reduction) at 60 min occurred in 79.4% (95% CI, 63.2–89.7%). In linear mixed-effects modeling, time remained a significant fixed effect (*p* < 0.001). **Conclusions**: Adjunctive GONB was associated with a rapid reduction in pain intensity in medically refractory APAC. These findings should be interpreted cautiously, given the uncontrolled design and concurrent treatment. Prospective controlled studies are warranted.

## 1. Introduction

Acute primary angle closure (APAC) is a vision-threatening ophthalmic emergency characterized by sudden obstruction of the anterior chamber angle with rapid elevation of intraocular pressure (IOP) [1,2]. Acute attacks are commonly accompanied by severe ocular pain, headache, nausea, and vomiting, and may lead to physiologic stress responses that complicate acute management—particularly in older adults with cardiometabolic comorbidities [3,4]. Prompt IOP reduction is essential to prevent optic nerve damage and irreversible visual loss [1,5].

Standard initial management consists of topical IOP-lowering medications and systemic agents (e.g., acetazolamide and hyperosmotic therapy), followed by definitive interventions such as laser peripheral iridotomy or early lens extraction depending on the clinical context [5,6]. However, intravenous mannitol may be contraindicated in patients with renal insufficiency or cardiovascular disease, and some eyes remain medically refractory despite maximal tolerated therapy [7]. In such cases, urgent surgical intervention, most commonly lens extraction, may be required [8].

In real-world practice, delays related to systemic evaluation, operating room availability, or medical stabilization may prolong the preoperative waiting period. During this interval, patients may continue to experience severe pain that limits cooperation and increases physiologic stress. Systemic opioids may provide short-term relief but carry risks of nausea, sedation, respiratory depression, and delirium in older adults [9,10]. Therefore, a targeted, rapid-onset, and low-systemic-burden analgesic strategy is clinically attractive.

In addition to patient discomfort, uncontrolled acute pain may interfere with clinical assessment, patient cooperation, and perioperative preparation, potentially complicating urgent surgical management. Despite this, structured analgesic approaches tailored to APAC-related pain during the preoperative period have not been well established.

The greater occipital nerve block (GONB) is a regional anesthetic technique widely used for migraine and other headache disorders [11,12,13]. Neuroanatomically, convergence of cervical (C2) and trigeminal afferents within the trigeminocervical complex provides a plausible basis for modulation of craniofacial and ocular nociception through occipital nerve blockade [14,15]. Despite its established use in headache medicine, evidence for GONB in APAC-related pain has not been reported [16,17,18].

Given its rapid onset of action and minimal systemic effects, GONB may represent a practical adjunctive option in acute ophthalmic settings where systemic analgesics are limited or contraindicated.

The clinical diagnosis and management framework of APAC in this study were interpreted in accordance with established ophthalmic reference standards and preferred practice patterns [19,20].

We therefore evaluated short-term changes in pain intensity and safety of adjunctive GONB in medically refractory APAC during the preoperative waiting period. In addition, we aimed to provide clinically relevant data on the feasibility and potential role of this approach in real-world perioperative management of APAC.

## 2. Methods

### 2.1. Study Design and Setting

This retrospective observational study was conducted at a tertiary referral center. Medical records of patients diagnosed with APAC between January 2017 and December 2024 were reviewed. The study setting reflects a real-world tertiary care environment in which patients with acute ophthalmic emergencies are managed collaboratively by ophthalmology and anesthesiology teams, particularly when urgent surgical intervention is required. The study adhered to the Declaration of Helsinki and was approved by the Institutional Review Board (DAUHIRB-24-213). The requirement for informed consent was waived because of the retrospective design.

### 2.2. Participants

Eligible patients met all of the following criteria: (1) clinical diagnosis of APAC confirmed by a glaucoma specialist, defined as acute ocular symptoms with IOP ≥ 30 mmHg at presentation; (2) severe headache documented in the record (NRS ≥ 5); and (3) requirement for urgent surgical intervention (lens extraction) due to contraindication to intravenous mannitol (e.g., renal impairment) and/or inadequate IOP control despite maximal tolerated topical and systemic medical therapy.

The diagnosis of APAC was based on a combination of clinical symptoms, elevated intraocular pressure, and anterior chamber angle findings assessed by slit-lamp examination and gonioscopy, as documented in the medical record.

Exclusion criteria included a history of chronic primary headache disorders, neurologic conditions associated with secondary headache, incomplete documentation of pain scores, or occipital nerve block within 3 months prior to presentation. Patients with insufficient documentation of the timing of pain assessments or procedural details were also excluded to ensure consistency of outcome evaluation.

### 2.3. Clinical Management of APAC

All patients received standard initial IOP-lowering therapy, including topical antiglaucoma medications and systemic treatment (oral acetazolamide and/or intravenous mannitol when not contraindicated).

Topical therapy typically included combinations of beta-blockers, alpha-agonists, and carbonic anhydrase inhibitors, administered according to institutional protocols and physician discretion.

Patients were considered medically refractory if IOP remained elevated or was deemed uncontrolled by the treating physician despite maximal tolerated therapy, prompting a decision for urgent lens extraction.

The decision for urgent surgery was made by the attending glaucoma specialist based on clinical judgment, taking into account IOP response, symptom severity, and systemic considerations.

During the interval between surgical decision and operating room transfer, GONB was performed for pain control at the physician’s discretion in patients with persistent headache despite ongoing APAC management. Because this decision was not protocolized, selection bias cannot be excluded.

GONB was generally considered when patients reported persistent moderate-to-severe headache despite ongoing medical therapy, particularly in situations where systemic analgesics were limited or undesirable due to potential adverse effects.

#### Greater Occipital Nerve Block Procedure

GONB was performed by a physician (SYP) experienced in regional anesthesia using a landmark-based technique. The injection site was identified approximately 2–3 cm lateral to the external occipital protuberance along the superior nuchal line. After aseptic preparation, 3–4 mL of 0.5% bupivacaine was injected around the greater occipital nerve.

A standard sterile technique was used in all cases, and the needle was advanced carefully to avoid vascular puncture. Negative aspiration was performed prior to injection to minimize the risk of intravascular administration.

The procedure was performed unilaterally or bilaterally according to headache distribution. Patients were observed for at least 30 min for immediate adverse events.

The procedure was performed at bedside without imaging guidance, reflecting routine clinical practice in an emergency setting. Basic monitoring, including vital signs and symptom assessment, was maintained during and after the procedure.

### 2.4. Outcome Measures

The primary outcome was the change in headache intensity measured using an 11-point NRS (0–10) from baseline (pre-block) to 60 min after GONB. Secondary outcomes included (1) NRS change at 30 min; (2) proportion achieving clinically meaningful pain relief (≥3-point NRS reduction); (3) ≥50% reduction from baseline; (4) mild pain intensity (NRS ≤ 3) at 60 min; and (5) documented procedure-related complications (e.g., hematoma, dizziness, infection, neurologic deficit).

Pain scores were obtained as part of routine clinical documentation and were recorded by clinical staff using a standardized numeric rating scale assessment.

Baseline NRS was defined as the pain score recorded immediately before GONB after initiation of standard APAC treatment and surgical decision-making. Follow-up NRS scores were recorded at approximately 30 and 60 min after GONB.

The timing of follow-up assessments was based on routine clinical monitoring intervals in the preoperative setting rather than a rigid research protocol.

Because all patients had already received standard APAC treatment prior to GONB, observed changes in pain scores reflect the combined clinical course and do not isolate the independent effect of GONB.

### 2.5. Statistical Analysis

Statistical analyses were performed using R software (version 4.2.2).

Continuous variables are presented as mean ± SD or median (IQR), and categorical variables as number (%). Normality was evaluated using the Shapiro–Wilk test.

Changes in NRS over time were assessed using a linear mixed-effects model with random intercepts for participants and time (baseline, 30 min, 60 min) as a fixed effect. Pairwise comparisons were adjusted using the Bonferroni correction. This modeling approach was selected to account for within-subject correlation and variability in repeated pain measurements over time.

Responder rates are presented with 95% CIs calculated using the Wilson method. Given the observational design and limited sample size, multivariable modeling and NNT estimation were not performed.

Statistical analyses were conducted with an emphasis on descriptive and longitudinal trends rather than causal inference.

All tests were two-sided, with *p* < 0.05 considered statistically significant.

## 3. Results

### 3.1. Patient Characteristics

Thirty-four patients with medically refractory APAC who received adjunctive GONB during the preoperative waiting period were included. The mean age was 68.4 ± 9.4 years, and 25 patients (73.5%) were women. Mean baseline IOP was 52.6 ± 9.0 mmHg. The median time from surgical decision to operating room transfer was 4.0 h (IQR, 3.0–5.8 h). Baseline, 30 min, and 60 min pain scores were available for all patients (Table 1). The cohort reflects an elderly population with acute disease severity, consistent with the clinical profile of patients typically requiring urgent intervention for APAC in tertiary care settings.

### 3.2. Pain Outcomes (Table 2, Figure 1 and Figure 2)

Baseline headache intensity was 7.8 ± 1.1 on the 11-point NRS. Mean NRS decreased to 4.1 ± 1.5 at 30 min and 3.6 ± 1.3 at 60 min. The mean reduction from baseline was −3.8 ± 2.0 points at 30 min and −4.2 ± 1.9 points at 60 min (both *p* < 0.001). The mean baseline-to-60 min change was −4.21 (95% CI, −4.88 to −3.54). Between 30 and 60 min, an additional mean reduction of −0.44 ± 0.61 points was observed (*p* = 0.0002). A progressive reduction in pain intensity was observed over time, with the largest decrease occurring within the first 30 min, followed by additional improvement at 60 min.

**Table 2:** Changes in Headache Intensity Following Greater Occipital Nerve Block.

Variable	Value
NRS at 30 min	4.1 ± 1.5
NRS at 60 min	3.6 ± 1.3
Mean change at 30 min (Baseline → 30 min)	−3.76 ± 2.02
Mean change at 60 min (Baseline → 60 min)	−4.21 ± 1.92
95% CI (baseline → 60 min)	−4.88 to −3.54
≥3-point reduction	27 (79.4%)
≥50% reduction	22 (64.7%)
NRS ≤ 3 at 60 min	16 (47.1%)

Values are presented as mean ± standard deviation or number (%). NRS = numerical rating scale; CI = confidence interval.

**Figure 1:**
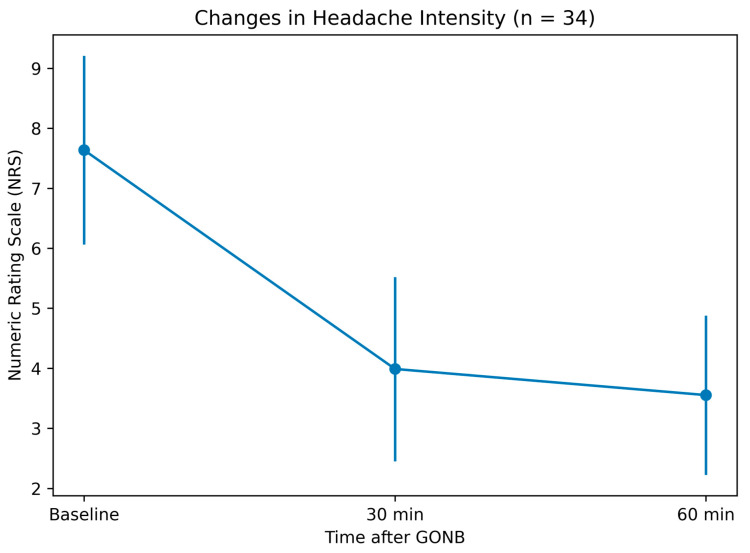
**Changes in headache intensity following greater occipital nerve block.** Mean Numeric Rating Scale (NRS) scores at baseline, 30 min, and 60 min after greater occipital nerve block (GONB) in patients with medically refractory acute primary angle closure (*n* = 34). Error bars represent standard deviation. Headache intensity decreased significantly at both 30 and 60 min compared with baseline (*p* < 0.001, linear mixed-effects model). A further significant reduction was observed between 30 and 60 min (*p* = 0.0002).

**Figure 2:**
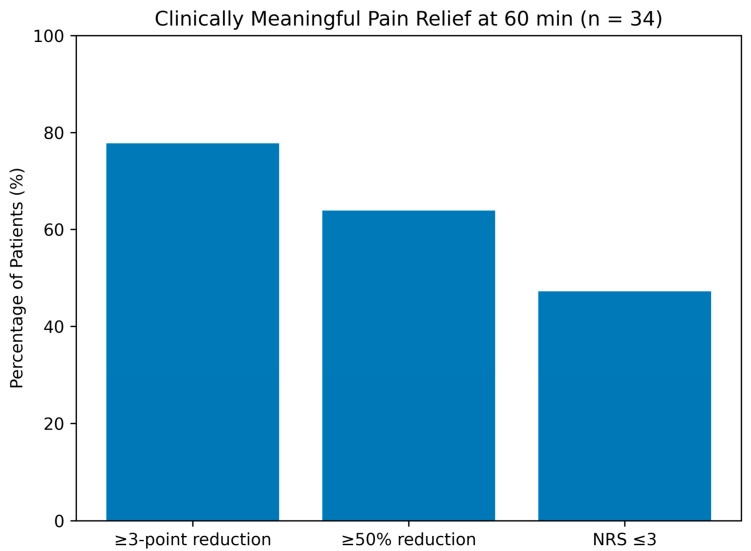
**Proportion of patients achieving clinically meaningful pain relief.** Bar graph showing the proportion of patients achieving a ≥3-point reduction in Numeric Rating Scale (NRS) score (79.4%), ≥50% reduction from baseline (64.7%), and mild pain intensity (NRS ≤ 3; 47.1%) at 60 min following greater occipital nerve block (GONB) in patients with medically refractory acute primary angle closure (*n* = 34). Error bars represent 95% confidence intervals calculated using the Wilson method.

### 3.3. Responder Analyses

Clinically meaningful pain relief (≥3-point reduction) at 60 min was achieved in 27 patients (79.4%; 95% CI, 63.2–89.7%). A ≥50% reduction from baseline occurred in 22 patients (64.7%; 95% CI, 47.9–78.5%), and 16 patients (47.1%; 95% CI, 31.5–63.3%) achieved mild pain intensity (NRS ≤ 3) at 60 min.

Patients with severe baseline pain (NRS ≥ 8; *n* = 22) demonstrated a comparable magnitude of reduction (−5.3 ± 1.2 points).

These responder analyses indicate that a substantial proportion of patients experienced clinically relevant improvement within a short time frame following the procedure.

### 3.4. Pain Trajectory Modeling

Linear mixed-effects modeling confirmed a significant effect of time on NRS reduction (*p* < 0.001). The model-estimated mean reduction at 60 min was −4.21 points (95% CI, −4.75 to −3.66). The consistency between observed mean changes and model-based estimates supports the stability of the observed temporal trend in pain reduction.

**Figure 3:**
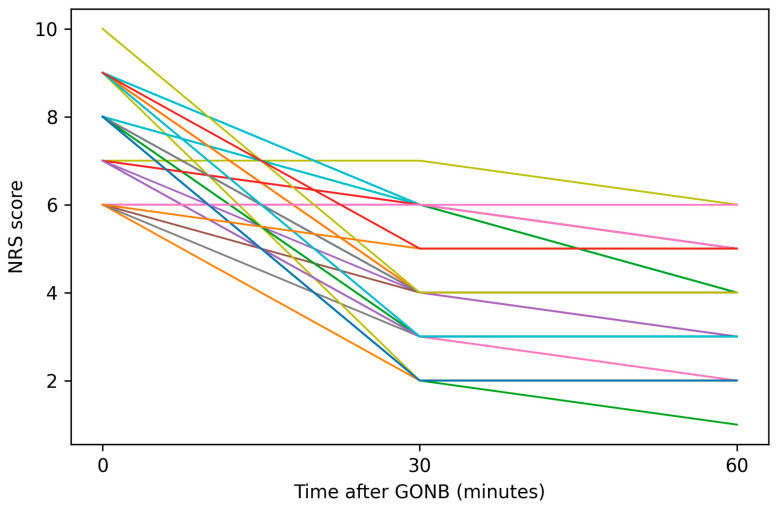
**Individual trajectories of headache intensity following greater occipital nerve block.** Line graph illustrating changes in Numeric Rating Scale (NRS) scores at baseline, 30 min, and 60 min after GONB in patients with medically refractory acute primary angle closure (*n* = 34). Each line represents an individual patient. Different colors are used for visual distinction only and do not represent specific subgroups. A consistent within-subject reduction in pain intensity was observed across the majority of patients.

Individual patient trajectories of NRS scores over time are illustrated in Figure 3, demonstrating consistent within-subject pain reduction across the majority of patients. Most patients demonstrated a monotonic decrease in NRS scores, with limited variability in the direction of response across individuals.

### 3.5. Safety

No serious procedure-related adverse events were documented. There were no recorded cases of hematoma, infection, or neurologic deficit during the observed interval. No patients required additional intervention related to the procedure, and no clinically significant hemodynamic or neurologic complications were reported.

## 4. Discussion

In this observational analysis, adjunctive GONB was associated with a reduction in headache intensity among patients with medically refractory APAC during the preoperative waiting period. The mean improvement exceeded four NRS points within 60 min, and 79.4% of patients achieved a ≥3-point reduction, a threshold commonly used to represent clinically meaningful acute pain relief [21,22]. The magnitude and rapid onset of pain reduction observed in this study are notable in the context of acute ophthalmic emergencies, where timely symptom control may have practical implications for both patient comfort and perioperative management.

The observed pain reduction is biologically plausible. Nociceptive afferents from ocular and craniofacial structures converge with upper cervical inputs within the trigeminocervical complex, a key relay in headache pathophysiology [14,17]. The greater occipital nerve originates primarily from the dorsal ramus of C2 and provides a peripheral access point for modulating this convergent system. In headache medicine, GONB has demonstrated short-term efficacy in migraine and chronic headache conditions, including randomized placebo-controlled settings [16,17,18]. Although APAC-related headache represents a secondary headache disorder, shared trigeminal nociceptive pathways and central sensitization mechanisms may contribute to the observed response [4,14]. These mechanisms support the concept that peripheral nerve blockade may influence centrally mediated pain pathways even in conditions where the primary pathology is ocular rather than neurologic.

Notably, the magnitude of pain reduction was not correlated with baseline IOP in our cohort, suggesting that observed pain reduction may be mediated primarily through neural modulation rather than indirect effects of IOP reduction. This observation is consistent with the concept that acute pain intensity in APAC may reflect both peripheral trigeminal activation and central processing, including sensitization and stress-related amplification [3,4]. This dissociation between IOP and pain intensity highlights the multifactorial nature of symptom generation in APAC and underscores the potential role of targeted analgesic strategies beyond pressure-lowering treatment alone.

Severe acute pain triggers sympathetic activation and neuroendocrine stress responses [3], which may be clinically relevant in older APAC patients with comorbidities. Systemic opioids, while effective, are associated with adverse outcomes in older hospitalized adults, including delirium and respiratory compromise [9,10]. In contrast, peripheral nerve blocks may provide targeted analgesia with minimal systemic exposure. Our absence of major complications aligns with prior reports supporting the overall safety of peripheral nerve blocks for headache when performed by experienced clinicians [11,18]. In this context, GONB may offer a pragmatic alternative in situations where systemic analgesics are contraindicated or poorly tolerated, particularly in elderly patients with multiple comorbid conditions.

From a pragmatic standpoint, GONB may be particularly useful when urgent surgery cannot be performed immediately due to system-level constraints. The procedure can be performed at the bedside with minimal equipment and does not interfere with subsequent ocular surgery. These features may facilitate workflow in tertiary emergency settings where APAC patients often require rapid stabilization and multidisciplinary coordination. The simplicity and rapid implementation of the procedure may also support its integration into acute care pathways, particularly in high-volume centers where delays in operating room access are common.

This study has limitations. The retrospective design and lack of a contemporaneous control group limit causal inference, and spontaneous pain fluctuation or regression to the mean cannot be excluded. All patients received concurrent APAC treatment prior to GONB; therefore, the independent effect of GONB cannot be isolated. The sample size was modest, and the observation window was limited to 60 min; therefore, the durability of analgesia beyond the immediate preoperative interval remains unknown. Additionally, physician discretion in performing GONB may introduce selection bias. Furthermore, procedural factors such as unilateral versus bilateral block and variation in clinical timing were not standardized, which may have contributed to variability in response. Finally, as this was a single-center study, the generalizability of the findings to other clinical settings may be limited.

These findings should be considered preliminary and hypothesis-generating. Prospective controlled studies are warranted to confirm these findings and to define optimal patient selection, procedural parameters, and comparative effectiveness versus standardized systemic analgesic regimens. Future studies should incorporate standardized protocols for GONB administration, including laterality and timing relative to medical therapy, and should evaluate outcomes in larger and more diverse patient populations. Randomized trials in emergency ophthalmic settings would be particularly informative. In addition, longer follow-up periods would be valuable to assess the duration of analgesic effects and their potential impact on perioperative outcomes.

## 5. Conclusions

Adjunctive GONB was associated with a reduction in headache intensity in medically refractory APAC during the preoperative waiting period. Longer follow-up periods would be valuable to assess the duration of analgesic effects and their potential impact on perioperative outcomes. These findings should be interpreted cautiously, given the uncontrolled design and concurrent treatment, and should be considered hypothesis-generating. Prospective controlled trials are warranted.

## Figures and Tables

**Table 1:** Baseline Clinical Characteristics of Patients With Medically Refractory Acute Primary Angle Closure.

Variable	Value
Age, years	68.4 ± 9.4
Female, *n* (%)	25 (73.5%)
Baseline IOP, mmHg	52.6 ± 9.0
Time from surgical decision to operating room transfer, hours	4.0 (3.0–5.8)
Baseline NRS score	7.8 ± 1.1

Values are presented as mean ± standard deviation or median (interquartile range) unless otherwise indicated. IOP = intraocular pressure; NRS = numerical rating scale.

## Data Availability

The datasets generated and analyzed during the current study are available from the corresponding author on reasonable request.
